# Treatment Buddies Improve Clinic Attendance among Women but Not Men on Antiretroviral Therapy in the Nyanza Region of Kenya

**DOI:** 10.1155/2016/9124541

**Published:** 2016-03-22

**Authors:** Charles Kibaara, Cinthia Blat, Jayne Lewis-Kulzer, Starley Shade, Patrick Mbullo, Craig R. Cohen, Elizabeth A. Bukusi

**Affiliations:** ^1^Family AIDS Care & Education Services (FACES), P.O. Box 614-40100, Kisumu, Kenya; ^2^Research Care and Training Program, Center for Microbiology Research, Kenya Medical Research Institute, Kisumu, Kenya; ^3^Department of Obstetrics, Gynecology and Reproductive Sciences, University of California San Francisco, San Francisco, CA 94143, USA

## Abstract

*Background.* Kenyan antiretroviral (ART) guidelines encourage treatment buddies (TBy) to maximize treatment adherence. This study examined the effect of TBys on clinic attendance in men and women on ART.* Methods.* This retrospective cohort study included all adult patients initiating ART from August 2007 to December 2011 at four health facilities in Kenya. Data were abstracted from electronic medical records and analyzed using Poisson regression.* Results.* Of 2,430 patients, 2,199 (91%) had a TBy. Relationship between TBy and clinic attendance differed in females and males (interaction *p* = 0.09). After demographic and clinic factor adjustment, females with a TBy were 28% more likely to adhere to all appointments than those without (adjusted aRR = 1.28; 95% CI 1.08–1.53), whereas males were no more likely to adhere (aRR = 1.01; 95% CI 0.76–1.32). Males reported partner/spouse (33%) or brother (11%) as the TBy while females reported sister (17%), partner/spouse (14%), or another family member (12%). Multivariable analysis found no association between clinic attendance and TBy relationship in either gender.* Conclusion.* Clinic attendance was higher among women with TBys but not men. Results support TBys to help women achieve ART success; alternate strategies to bolster TBy benefits are needed for men.

## 1. Introduction

In 2013, over 7.6 million HIV-infected Africans were receiving antiretroviral drugs [1], including approximately 650,000 in Kenya [2]. Regular clinic attendance is essential for monitoring and responding to disease progression in this population; providing prophylactic medications for opportunistic infections; sustaining drug adherence; and evaluating patients for drug toxicities. Patients who miss visits are at increased risk for interruptions in antiretroviral therapy (ART), poorer immunological and virological outcomes, and higher rates of loss to follow-up and mortality [3–9]. They are also more likely to experience HIV progression [10], viral resistance to drugs [7], and treatment failure [7]. Appointment adherence early in antiretroviral treatment may be particularly critical to long-term outcomes because side effects are more likely to occur when patients are initiated on a regimen [11, 12]. Although high clinic attendance has been achieved in some resource-limited settings [13], there remains an urgent need for practical interventions to improve attendance to avoid drug resistance and treatment failure.

Clinic attendance may be influenced by patient social contexts and interpersonal relations with family members and within communities. Social support systems can help patients overcome structural and financial barriers to accessing care, provide a context for patients to discuss concerns about their diagnosis and treatment, and buffer against community stigma [14]. Studies have found patients with social support are less likely to miss a clinic visit [15] and report an easier time reengaging in care after an appointment has been missed [16]. One strategy to utilize the benefits of social support recommended by Kenya's National AIDS and STI Control Program (NASCOP) is engagement of a patient-nominated treatment buddy (TBy), an individual, usually a trusted family member or friend, who commits to supporting the patient on ART. The TBy may accompany the patient to the clinic and help with treatment adherence [17, 18], provide lay counsel and encouragement, and support patients in establishing healthy behaviors, such as curbing drug and alcohol use [19].

Previous studies of TBys have focused largely on ART adherence and HIV outcomes, with mixed results. A prospective cohort study among patients initiating ART at public facilities in South Africa found higher odds of treatment success (defined as viral load < 400 copies/mL, CD4 ≥ 200 cells/mm^3^) at six months, one year, and two years after ART initiation among patients with TBys [20]. However, a randomized controlled trial (RCT) also in South Africa showed no difference in CD4 cell count or viral load at one or two years after ART initiation among patients who received directly observed ART (DOT-ART) by a TBy compared to a control group who self-administered ART [21], although survivorship was unexplainably better in the TBy DOT-ART group. A RCT in Nigeria found a significantly higher proportion of those in the TBy arm maintained timely ART drug pickup but showed no durable effect on CD4 cell count or mortality [22].

To our knowledge, only one other study has evaluated effects of a TBy on clinic attendance. A recent RCT of a TBy intervention among 174 patients attending an HIV clinic in Uganda found no difference in clinic attendance during the 28-week follow-up period [23]. Our study sought to reevaluate this finding within the context of HIV care in Kenya and consider the impact of the intervention separately for men and women. There is considerable evidence for gender differences in HIV health behaviors among African men and women: men have lower compliance with antiretroviral therapy [24, 25] and higher rates of attrition from care [25, 26] than women, which in turn contribute to their poorer immunological response to treatment and elevated risk of mortality [25, 27]. Although these disparities remain poorly understood, it appears that existing strategies to promote retention and adherence have been less successful in men than women. Based on these findings we sought to determine (1) the effect of TBy on clinic attendance within the first six months on ART in a cohort of HIV-infected male and female patients in Kenya and (2) whether this effect differs between men and women. In addition, because qualitative studies have also highlighted the influence of the relationship between TBys and patients [28, 29], we also sought to characterize the types of relatives and patients chosen as a TBy and explore whether choice of relative impacted clinic attendance in our study population.

## 2. Materials and Methods

### 2.1. Study Design and Participants

This was a retrospective cohort study of HIV-infected adult patients ≥15 years of age initiating ART between August 1, 2007, and December 23, 2011. The study targeted four Family AIDS Care & Education Services (FACES) supported facilities (PandiPieri, Lumumba, Family Health Options Kenya (FHOK), and Tuungane) in Kisumu County, where the prevalence of HIV (19%) is more than three times the national average for Kenya (6%) [30]. FHOK specializes in treatment of female sex workers, Tuungane serves pediatric and adolescent clients, and PandiPieri and Lumumba are high volume sites enrolling patients of all ages. Only patients engaged into HIV care on or after July 23, 2007 (following onset of electronic medical records capture), who had documented whether or not they had a TBy and who did not discontinue from care during the six-month post-ART follow-up were included in the study sample. This was done to establish the effect of the TBy on clinic attendance separate from the effect of the TBy on retention in care. Eligibility guidelines for ART initiation were revised during the study period from a CD4 count of ≤200 cells/mm^3^ to ≤350 cells/mm^3^ in 2011 following a change in national guidelines [31].

### 2.2. Pre-ART Clinic Procedures

Prior to ART initiation, the patient is counseled to disclose his/her status to one or two trusted individuals who can support his/her treatment, typically a partner, parent, daughter, sister, brother, friend, or neighbor. The patient brings the TBy to the clinic to participate in the pre-ART adherence counseling process, a series of three counseling and education sessions that cover HIV transmission and risk factors; disease progression and CD4 monitoring; purpose, benefits, and importance of ART medication and adherence; consequences of poor adherence; treatment schedule and reminder systems; and disclosure and positive living. TBys provide ongoing support for positive living and medication adherence. Patients are expected to notify the facility if there is a TBy change.

### 2.3. Clinic Attendance

In Kenya, a patient's first six-month observation period following ART initiation typically includes a first clinic visit two to four weeks after ART initiation and monthly thereafter. Providers assess health status, adherence, drug regimen tolerance, and toxicity and modify the ARV regimen if needed. Patients are provided with ARV refills to last them until their next clinic visit, which is scheduled that day. A patient may also come to the clinic between scheduled visits as needed. TBys may attend clinic appointments with the patient or may come in the patient's place to pick up the drugs.

### 2.4. Data Collection

Patient demographic factors, TBy status, and appointment adherence data were extracted from medical encounter forms collected during routine patient care and entered into an open source electronic medical records system database (OpenMRS system version 1.8.3). For the majority of patients (2416/2430), TBy details were extracted from the pre-ART adherence counseling form completed immediately prior to ART initiation. Another 14 patients whose TBy details could not be ascertained from the pre-ART adherence counseling form but who indicated having a TBy at the time of engagement into care were coded as having a TBy. Clinic appointment adherence was defined as completing all scheduled clinic appointments within the first six months on ART. An appointment was deemed completed if the patient was seen on or within three business days after the scheduled return date or had appeared in the clinic prior to the scheduled return date.

### 2.5. Measures and Statistical Analysis

The outcome was the proportion of patients who attended all clinic visits within the first six months on ART. TBy was operationalized as dichotomous for primary analyses (1 = having TBy; 0 = having no TBy) and categorical for a secondary analysis that examined clinic attendance by relationship with TBy (1 = having no TBy; 2 = partner is the TBy; 3 = another family member or friend is the TBy). Chi-square tests were performed to assess differences in demographic and clinical characteristics by TBy status. Multivariable Poisson regression with robust standard errors was used to obtain adjusted relative risks. An initial regression model with terms for TBy (dichotomous), gender, and their interaction found a significant difference in male and female response to treatment support at *p* < 0.20; thus we decided to develop separate models for males and females. Patient's age, marital status, year initiated on ART, CD4 count, and clinic site were included in each multivariable model on prior belief that they might confound the TBy relationship with clinic attendance. Educational status, WHO HIV clinical stage, and months from engagement in care until ART initiation were maintained in the multivariable models if associated with clinic attendance at *p* < 0.20 on bivariable analysis. Educational status did not meet this criterion in either men or women and was omitted from multivariable models. Data was analyzed using SAS version 9.3 (SAS Institute, Cary, NC).

### 2.6. Ethical Statement

Use of the program data for research was approved by the Kenya Medical Research Institute (KEMRI) Ethical Review Committee and the Committee on Human Research (11-05348) at the University of California San Francisco. All patient data were stripped of any identifying details and identified only by a unique patient number.

## 3. Results and Discussion

### 3.1. Sample Characteristics

Of 2,430 patients who met inclusion eligibility requirements in this study, 2199 (90.5%) had a TBy (Table 1). The majority of patients were female, had no more than a primary school education, and were married or partnered. Over 40% had been engaged in care for less than a month when initiated on ART and most had a CD4 cell count of <200 (prior to change in Kenyan National Guidelines) or <350 cells/mm^3^ at time of ART initiation. A greater proportion of males had a TBy than females, and a greater proportion of married/partnered patients had a TBy than single patients. Patients with a TBy were older. Proportion with a TBy was higher among patients at WHO stages 3 and 4 at initiation (Table 1).

On univariate analysis, patients with a TBy were more likely to achieve consistent appointment adherence (Table 2). The majority (60%) of patients with a TBy met all scheduled appointments during the six-month follow-up period, compared to just under half (49%) of those without a TBy [RR = 1.23; 95% CI 1.07–1.41]. The relationship between TBy and clinic attendance differed by gender (interaction *p* = 0.09). Equal proportions of men with and without a treatment buddy adhered to their visit schedule (57% versus 57%; RR = 1.00; 0.76–1.31). In females, 61% with a TBy consistently met scheduled appointments versus 47% without a TBy (RR = 1.31; 95% CI 1.11–1.53).

After adjusting for age, marital status, year initiated ART, months from engagement in care to ART initiation, CD4 count, WHO stage, and clinic site, females with a TBy were nearly 30% more likely to meet all scheduled appointments during the appointment follow-up period than those without one (adjusted aRR = 1.28; 95% CI 1.08–1.53; Table 3). Clinic attendance showed a positive association with increasing age, with adjusted risk significantly higher among females aged 30–44 than females aged 15–29. Being in a partnership, belonging to a more recent ART initiation cohort (2011 versus 2007, 2008, or 2009), and initiating ART at an earlier clinical WHO stage were all independently associated with better clinic attendance in females. Females seen at Tuungane, PandiPieri, or FHOK were less likely to have adhered to the appointment schedule than those at Lumumba.

As with the univariate results, TBy showed no association with clinic attendance in males after adjustment for age, marital status, year initiated ART, months from engagement in care to ART initiation, CD4 count, WHO stage, and clinic site (aRR = 1.01; 95% CI 0.76–1.32; Table 3). Clinic attendance was lower in males who initiated ART at WHO stages 3 and 4 compared to WHO stage 1. Clinic attendance was lower among males at Tuungane or PandiPieri compared to males at Lumumba.

### 3.2. Treatment Buddy Relationship to Patient

Type of relationship with the TBy differed by patient gender (*p* < 0.0001; data not shown). Males most commonly reported a partner/spouse (33%), brother (11%), or another family member (7%) as their TBy. Females more often reported the TBy to be a sister (17%), followed by the partner/spouse (14%) or another family member (12%). Relationship with TBy was missing for 28% of males (*n* = 217) and 28% of females (*n* = 397). On crude analysis, females whose TBy was a partner or spouse were more likely to have attended all appointments during the follow-up period than women whose TBy was another family member or friend (70% versus 61%; Table 4). However, after adjustment for age, marital status, year ART initiated, CD4 count, clinic site, months from engagement in care to ART initiation, and WHO stage, there was no longer a significant difference in clinic attendance by relationship with TBy among females (aRR = 1.01; 95% CI 0.90–1.14). Similarly, on crude analysis more males with a partner or spouse as the TBy met their appointment schedule than those with another family member or friend (62% versus 52%), but there was no significance difference in clinic attendance by relationship type with the TBy after multivariable adjustment (aRR = 1.05; 95% CI 0.89–1.24).

### 3.3. Discussion

In this study we found better clinic attendance among women with a TBy but not men. Women with a TBy were nearly 30% more likely to have kept all appointments within the first six months on therapy than women without a TBy. Clinic attendance was not significantly different in men with or without a TBy.

For women, these findings have promising implications. Clinically, consistent attendance is essential for timely ART delivery and monitoring for toxicities and treatment failure; preventing ART interruptions is crucial because treatment lapses can result in drug resistance and increased mortality risk [32]. Although we could not verify that ARV adherence or clinical outcomes were better in women with more consistent attendance, because the sites participating in this study follow a protocol of prescribing ART refills to last until the next visit, missed visits represented likely gaps in treatment, placing patients at risk for poorer HIV outcomes [5] and higher risk of drug resistance [7].

For men, our finding of no TBy effect on attendance is of special concern because men show a broader pattern of poorer engagement and retention [33], are known to delay HIV testing and enrollment in care compared to women, and are more likely to lapse in ARV adherence and drop out of care [32, 34]. While the reasons for these disparities require further study, recent qualitative work by Chikovore et al. highlights the importance of social role definitions which place greater responsibility for family income generation on men [35]. Within a context of job scarcity and constant insecurity, pressure to find and maintain work may lead men to relegate health considerations. A retrospective analysis of clinical data from HIV care facilities in Kenya also found work commitments the most common reason for missed visits among men, while women were more likely to report family commitments [34]. With respect to our own findings, we speculate that TBys may be better able to help with competing childcare and household obligations than occupational commitments or costs of lost wages and contracts faced by workers who take time off.

Gender differences in social norms and economic standing may also influence how men and women relate to the TBy in other ways. Norms of masculinity that emphasize resilience and self-reliance discourage African men from seeking help from others when needed [36]. At the same time, the lower economic status of African women may lead to greater dependency on community support to surmount hardships and therefore indirectly promote better attendance behavior. A recent ethnographic study conducted in Nigeria, Tanzania, and Uganda found that HIV patients adhere to treatment guidelines to fulfill the expectations of members of their social network who provide financial or practical support [37]. In resource-poor environments, patients must borrow money from friends and family for food, transport to clinic, and other needs. Adherence demonstrates that the patient will use the investment responsibly and can be relied upon with future resources for care-related or other expenses. As such, we might expect TBys to exert greater influence over more marginal and economically vulnerable patients.

The protocols followed at the clinics included in this study allowed patients to self-select the TBy from among trusted family and friends. Treatment buddies participated in pre-ART preparatory education and counseling sessions with the patient and supported patients with medication reminders. TBys were also encouraged to attend clinic appointments with patients if possible and support patients with pickup of ART refills when necessary and feasible. To our knowledge, only one other study has evaluated a similar patient self-selected TBy intervention on clinic attendance, a recent RCT led by Kunutsor et al. among 174 patients on ART at a hospital in Uganda [23]. That study, which pooled together results for men and women, found a weak but not statistically significant trend towards better clinic attendance in the TBy group compared to the standard-of-care group. It may be that the larger sample included in this study allowed for better detection of differences between groups or that the decision to stratify by gender highlighted an effect among women masked in the earlier trial.

Previous research has shown that patients and providers choose treatment buddies who are confidantes and able to influence health-related decision-making [25, 28, 37]. Trust, geographic proximity to the TBy, emotional availability, reciprocity, and material resources are all important features of the treatment buddy-patient relationship [29]. In our study, most patients selected a family member to serve as a TBy. Women most often chose a sister, perhaps because they perceived sisters to be more supportive than other family members [38]. Men were more than twice as likely to report the partner as the TBy than women, probably because they faced less risk of partner violence or stigma. Relationship type did not however appear to influence the effect of the TBy on clinic attendance in either gender. For women, clinic attendance was higher whether they chose the partner or another family member or friend. In men, clinic attendance was not significantly different whether they chose the partner, another family member or friend, or no TBy.

Strengths of our study include a large, representative cohort of patients at multiple HIV care settings in Kenya. This is the first study to our knowledge to examine the effectiveness of TBys as an intervention in Kenya and the only large-scale study to examine type of relationship to the TBy among HIV patients in sub-Saharan Africa. A study limitation was that it was based on observational data and may be subject to unmeasured confounding. In particular, because of the self-nominated nature of the TBy intervention at FACES-supported clinics, patients with a TBy may have generally stronger social connections and greater support within their social network than those without a TBy. Thus the TBy may serve as a marker for social capital. Patients initiated on ART without a TBy also reflect an unusual exception to Kenyan guidelines that encourage the selection of a TBy and may therefore differ from the general population of new patients on ART. For example, they may be more disadvantaged or have more clinically advanced illness. Nevertheless, we were able to control for a number of sociodemographic and disease markers to help account for some of these differences. A second limitation of this study was that TBy status could only be ascertained for the 48% of patients initiated on ART during the study period for whom pre-ART adherence counseling data was available in the administrative database. Third, we were not able to directly associate clinic attendance with ARV adherence or clinical outcomes. However, the relationship between clinic attendance and ART adherence and outcomes has been firmly established [3–5, 8]. Finally, we only examined attendance patterns during the first six months on ART. One- and two-year follow-up are needed to assess longer-term durability of our findings.

## 4. Conclusions

In summary, clinic attendance was higher during the first six months of ART among women with TBys but not men. These results support TBys to help women achieve ART success in resource-poor settings; alternate strategies to bolster TBy benefits are needed for men. Specialized trainings for TBys and caregiver support groups may further enhance the impact of TBys on adherence and health outcomes for all patients on ART.

## Figures and Tables

**Table 1:** Sample characteristics (*n* = 2430)^*∗*^.

Characteristics	TBy (*n* = 2199)	No TBy (*n* = 231)	*p* value^*∗∗*^
Gender			<0.0001
Male	770 (94.8)	42 (5.2)	
Female	1429 (88.3)	189 (11.7)	
Age (years; enrollment), median (IQR)	32 (27–39)	30 (25–35)	<0.0001
15–29	852 (88.3)	113 (11.7)	
30–44	1038 (90.7)	107 (9.3)	
45+	309 (96.6)	11 (3.4)	
Education level (enrollment)			0.05
None	57 (98.3)	1 (1.7)	
Primary	1118 (91.2)	108 (8.8)	
Secondary	665 (89.4)	79 (10.6)	
College	164 (93.7)	11 (6.3)	
Missing	195 (85.9)	32 (14.1)	
Marital status (enrollment)			<0.0001
Single	894 (87.9)	123 (12.1)	
Partnered	1076 (93.1)	80 (6.9)	
Missing	229 (89.1)	28 (10.9)	
Children in household (enrollment)			0.11
0	469 (90.9)	47 (9.1)	
1	447 (91.4)	42 (8.6)	
2	457 (88.1)	62 (12.0)	
3+	563 (92.1)	48 (7.9)	
Missing	263 (89.2)	32 (10.9)	
Year ART initiated			<0.0001
2007	31 (79.5)	8 (20.5)	
2008	242 (80.9)	57 (19.1)	
2009	513 (91.3)	49 (8.7)	
2010	749 (92.5)	61 (7.5)	
2011	664 (92.2)	56 (7.8)	
Months from enrollment to ART initiation, median (IQR)	1 (0–3)	1 (0–6)	0.03
<1	964 (91.7)	87 (8.3)	
1-2	530 (91.7)	48 (8.3)	
2-3	131 (86.2)	21 (13.8)	
>3	574 (88.4)	75 (11.6)	
CD4 count (cells/mm^3^; ART initiation), median (IQR)^†^	180 (82–260)	189 (113–280)	0.52
>350	155 (88.6)	20 (11.4)	
200–350	797 (90.2)	87 (9.8)	
<200	1207 (91.0)	119 (9.0)	
WHO stage (ART initiation)^†^			0.03
1	526 (88.9)	66 (11.2)	
2	697 (88.7)	89 (11.3)	
3	809 (92.7)	64 (7.3)	
4	156 (92.9)	12 (7.1)	
Clinic site			<0.0001
Lumumba	993 (90.8)	101 (9.2)	
Tuungane	73 (97.3)	2 (2.7)	
PandiPieri	1011 (94.9)	54 (5.1)	
FHOK	122 (62.2)	74 (37.8)	
Retention during first 6 months on ART			0.001
Missed ≥1 visits	890 (88.2)	119 (11.8)	
Missed no visits	1309 (92.1)	112 (7.9)	

^*∗*^ *n* (%) shown unless otherwise indicated.

^*∗∗*^Significance tests based on nonmissing values.

^†^There were missing data for the following predictors:

CD4 count: 45 patients; 40 with treatment buddy and 5 without treatment buddy.

WHO stage: 10 patients, all with treatment buddy.

**Table 2:** Unadjusted relative risks for factors associated with clinic attendance during the first six months on ART, overall, and by gender.

Predictor	Total (*n* = 2430)			Male (*n* = 812)			Female (*n* = 1618)		
	*n*	% attended	RR (95% CI)	*n*	% attended	RR (95% CI)	*n*	% attended	RR (95% CI)
Has treatment buddy									
Yes	1309	59.5	1.23 (1.07–1.41)	770	57.1	1.00 (0.76–1.31)	1429	60.8	1.31 (1.11–1.53)
No	112	48.5	REF	42	57.1	REF	189	46.6	REF
						Treatment buddy × gender interaction *p* value = 0.09			

**Table 3:** Adjusted relative risks for factors associated with clinic attendance during the first six months on ART, by gender.

Predictor	Male (*n* = 716)					Female (*n* = 1405)				
	*n*	% attended	aRR	95% CI	*p* value	*n*	% attended	aRR	95% CI	*p* value
Has treatment buddy										
Yes	679	57.6	1.01	0.76–1.32	0.97	1243	60.6	1.28	1.08–1.53	0.005
No	37	59.5	REF			162	45.1	REF		
Age										
15–29	173	56.7	REF			681	54.9	REF		
30–44	394	56.6	0.99	0.85–1.15	0.90	599	62.4	1.12	1.03–1.22	0.01
45+	149	61.7	1.05	0.87–1.25	0.62	125	62.4	1.14	0.98–1.33	0.08
Marital status										
Single	191	53.9	REF			798	54.0	REF		
Partnered	525	59.1	1.02	0.89–1.18	0.75	607	65.1	1.10	1.01–1.20	0.04
Year ART initiated										
2007	6	16.7	0.20	0.03–1.24	0.08	26	42.3	0.62	0.39–0.99	0.04
2008	78	59.0	0.83	0.66–1.03	0.09	151	54.3	0.80	0.68–0.94	0.007
2009	163	60.7	0.97	0.82–1.14	0.71	318	53.8	0.83	0.73–0.94	0.003
2010	245	57.1	0.90	0.77–1.05	0.17	479	61.0	0.94	0.85–1.04	0.24
2011	224	56.7	REF			431	62.7	REF		
Months from engagement in care to ART initiation										
<1	354	52.5	REF			581	58.5	REF		
1-2	188	60.6	1.07	0.92–1.23	0.37	315	55.9	0.92	0.82–1.04	0.18
2-3	42	61.9	1.12	0.85–1.48	0.40	96	51.0	0.88	0.72–1.08	0.21
>3	132	65.9	1.12	0.94–1.32	0.20	413	63.2	1.04	0.93–1.16	0.45
CD4 count										
>350	38	65.8	1.09	0.84–1.40	0.51	115	56.5	0.92	0.77–1.09	0.32
200–350	214	63.1	1.04	0.90–1.20	0.61	565	63.4	1.05	0.95–1.16	0.36
<200	464	54.5	REF			725	55.6	REF		
WHO stage										
1	151	67.6	REF			365	66.0	REF		
2	194	61.9	0.93	0.80–1.09	0.36	506	58.1	0.91	0.83–1.01	0.09
3	308	51.3	0.80	0.69–0.93	0.003	454	57.1	0.91	0.81–1.01	0.08
4	62	51.6	0.74	0.57–0.96	0.03	80	40.0	0.65	0.49–0.85	0.002
Clinic site										
Lumumba	345	72.5	REF			577	71.4	REF		
Tuungane	22	45.5	0.58	0.36–0.92	0.02	41	31.7	0.44	0.28–0.69	0.0003
PandiPieri	349	43.8	0.58	0.51–0.67	<0.0001	610	52.3	0.69	0.63–0.76	<0.0001
FHOK	na	na	na	na	na	177	46.3	0.68	0.57–0.81	<0.0001

**Table 4:** Adjusted relative risks for factors associated with clinic attendance during the first six months on ART including relationship to TBy, by gender.

Predictor	Male				Female			
	*n*	% attended	RR (95% CI) (*n* = 595)	aRR (95% CI)^*∗*^ (*n* = 535)	*n*	% attended	RR (95% CI) (*n* = 1221)	aRR (95% CI)^*∗*^ (*n* = 1091)
Treatment buddy relationship								
Partner/spouse	250	62.4	1.18 (1.02–1.36)	1.05 (0.89–1.24)	198	69.7	1.14 (1.03–1.27)	1.01 (0.90–1.14)
Other	303	51.8	REF	REF	834	60.9	REF	REF
None	42	57.1	1.08 (0.82–1.44)	1.03 (0.77–1.37)	189	46.6	0.76 (0.65–0.90)	0.75 (0.62–0.90)

^*∗*^Adjusted for age, marital status, year ART initiated, CD4 count, clinic site, months from engagement in care to ART initiation, and WHO stage.
